# Serial blood eosinophils and clinical outcome in patients with chronic obstructive pulmonary disease

**DOI:** 10.1186/s12931-018-0840-x

**Published:** 2018-07-13

**Authors:** Sun Hye Shin, Hye Yun Park, Danbee Kang, Juhee Cho, Sung Ok Kwon, Joo Hun Park, Jae Seung Lee, Yeon-Mok Oh, Don D. Sin, Woo Jin Kim, Sang-Do Lee, KOLD Study Group

**Affiliations:** 1 Division of Pulmonary and Critical Care Medicine, Department of Medicine, Samsung Medical Center, Sungkyunkwan University School of Medicine, 81 Irwon-ro, Gangnam-gu, Seoul, 06351 South Korea; 2 Departments of Health Sciences and Technology and Clinical Research Design and Evaluation, Samsung Advanced Institute of Health Sciences and Technology, Sungkyunkwan University, Seoul, South Korea; 3 Cancer Education Center, Samsung Comprehensive Cancer Center, Samsung Medical Center, Sungkyunkwan University School of Medicine, Seoul, South Korea; 4 Departments of Health, Behavior, and Society and Epidemiology, Johns Hopkins Bloomberg School of Public Health, Baltimore, MD USA; 5 Biomedical Research Institute, Kangwon National University Hospital, Chuncheon, South Korea; 6 Department of Pulmonary and Critical Care Medicine, Ajou University School of Medicine, Suwon, South Korea; 7 Department of Pulmonary and Critical Care Medicine, Clinical Research Center for Chronic Obstructive Airway Diseases, Asan Medical Center, University of Ulsan College of Medicine, Seoul, South Korea; 8 Respiratory Division, Department of Medicine, University of British Columbia, Vancouver, BC Canada; 9 Department of Internal Medicine and Environmental Health Center, Kangwon National University Hospital, School of Medicine, Kangwon National University, 156 Baengyeong-ro, Chuncheon-si, Gangwon-do 200-722 South Korea

**Keywords:** Chronic obstructive pulmonary disease, Eosinophil, Stability, Lung function

## Abstract

**Background:**

Blood eosinophils have been suggested as a potential biomarker in chronic obstructive pulmonary disease (COPD), and their stability over time has been investigated in a few studies. However, the association between the stability of blood eosinophils and long-term clinical outcomes in COPD patients has yet to be fully elucidated. This study aimed to evaluate the stability of blood eosinophils and its association with clinical outcomes in COPD patients.

**Methods:**

In total, 299 COPD patients from the Korean Obstructive Lung Disease cohort with at least two blood eosinophil measurements were included. Patients were stratified according to a cut-off of 300 cells/μL, and the association between eosinophil changes and all-cause mortality was analysed. The annual decline in forced expiratory volume in 1 s (FEV_1_), serial changes in St George’s Respiratory Questionnaire score (SGRQ), and exacerbations during follow-up were compared among eosinophil groups.

**Results:**

Patients were stratified into three groups according to the blood eosinophil cut-off: persistently < 300 cells/μL (PL; *n* = 175), variable (V; *n* = 68), and persistently ≥300 cells/μL (PH; *n* = 56). There were no significant differences in baseline characteristics (age, sex, smoking, body mass index, use of inhaled corticosteroids, exacerbations in the previous year, FEV_1_ (L or % predicted), or emphysema score) among the groups. During a median follow-up of 6.0 years, the PH group had a better survival rate than the PL group (adjusted mortality rate ratio, 0.29; 95% confidence interval, 0.09–0.97; *P* = 0.045). The PH group also showed improved symptoms and impact domains of SGRQ score compared to the PL group. No difference was found in annual FEV_1_ decline or exacerbations during follow-up among the groups.

**Conclusions:**

Patients with persistently high blood eosinophils had a better survival rate than those with persistently low blood eosinophils. Serial follow-up of blood eosinophils could help to predict outcomes in COPD patients.

**Electronic supplementary material:**

The online version of this article (10.1186/s12931-018-0840-x) contains supplementary material, which is available to authorized users.

## Background

Neutrophilic inflammation profiles are generally recognised in chronic obstructive pulmonary disease (COPD) [1] but eosinophilic inflammation in sputum has also been investigated as a predictor of the corticosteroid response [2, 3]. Recently, awareness of the role played by peripheral blood eosinophils in COPD has increased, with reports showing that the peripheral blood eosinophil count corresponds to eosinophilic inflammation in the airway [4–6]. Although there is no consensus regarding the most appropriate cut-off value, higher blood eosinophils are linked to an improved response to inhaled corticosteroids (ICS) in preventing lung function decline [7] and exacerbations in COPD patients [8–12]. Together alongside concerns regarding the pneumonia risk associated with ICS [13] and the efficacy of dual bronchodilator therapy [14, 15], the Global Initiative for Chronic Obstructive Lung Disease suggests the use of ICS in frequent exacerbators with high blood eosinophils. [16] However, clinical outcomes related to increased blood eosinophils show heterogeneous features: increased risk of acute exacerbations (AE) [17] and readmission [18], but with fewer symptoms [19], better baseline lung function [19] with slower decline [20], fewer comorbidities [21] and an even lower mortality rate [22, 23].

As a biomarker in COPD, a single blood eosinophil measurement might not indicate a stable condition. ECLIPSE (Evaluation of COPD Longitudinally to Identify Predictive Surrogate End-points) investigators found that only 51% of subjects had a stable eosinophil count of below or above 2% over a 3-year period [19]. Another prospective observational study that measured blood eosinophils every 3 months over a 1-year period revealed that 65% of COPD patients remained persistently above or below a level of 400 cells/μL [24]. Recently published data from the general population showed that the long-term stability of blood eosinophils using a cut-off of 340 cells/μL was 75% across 1 year, 49% across 4 years, and 35% across 8 years [25]. That study also found significantly lower stability in patients with a baseline blood eosinophil count greater than 340 cells/μL. Therefore, despite variable cut-off values, the results of previous studies suggest that a considerable proportion of patients will have oscillating blood eosinophil levels over time. Nevertheless, a lack of data precludes comparisons between clinical outcomes and the stability of blood eosinophils over time. Therefore, we evaluated blood eosinophil stability across two measurements and sought associations with long-term clinical outcomes, such as lung function decline and mortality, in COPD patients using data from an observational cohort study.

## Methods

### Study population

The study participants included 466 COPD patients from the Korean Obstructive Lung Disease (KOLD) cohort. Details of the KOLD study protocol were published previously [26]. Recruitment took place in pulmonary clinics across 16 hospitals in the Republic of Korea from June 2005 to June 2015. The inclusion criteria were: 1) 40 years of age or older; 2) COPD, defined as a post-bronchodilator forced expiratory volume in 1 s (FEV_1_)/forced vital capacity (FVC) ratio < 0.7 and a smoking history of more than 10 pack-years; and 3) no history or radiographic evidence of tuberculosis, bronchiectasis, or other pulmonary disorders. Participation was further restricted to those with baseline and serial peripheral blood eosinophil counts; one patient with only a baseline spirometry measurement was excluded. The final sample included 299 patients. The study protocol was approved by the Institutional Review Board of Asan Medical Center (no. 2005–0010). Written informed consent was obtained from all participants.

### Blood eosinophils

Using whole blood samples collected from patients, complete blood cell counts were conducted using an automated hematology analyser at each participating hospital. Samples were taken at baseline and every year, where possible, as per the protocol. Two serial measurements, baseline and at 1 year (*n* = 278), baseline and at 2 years due to the absence of 1-year measurement (*n* = 13), or baseline and at 3 years due to an absence of 1- and 2-year measurements (*n* = 8) were used in this analysis. The percentage of eosinophils was multiplied by the white blood cell count to give the absolute eosinophil count. A pre-specified eosinophil cut-off of 300 cells/μL was used to characterise the study cohort and determine associations with clinical outcomes [10, 20, 27]. Using this cut-off, the subjects were stratified into three groups according to the serial eosinophil count: persistently < 300 cell/μL (persistently low; PL), variable above and below 300 cell/μL (variable; V), and persistently ≥300 cell/μL (persistently high; PH).

### Outcome measurements

The primary endpoint was the mortality rate at 8 years. Participants were followed from their baseline visit to death or the last available visit.

As per the study protocol, spirometry was performed every 3 months where possible. The mean (standard deviation, SD) number of spirometry tests per subjects was 9.1 (4.6) and the mean (SD) interval between each spirometry test was 0.56 (0.36) years. Spirometry was performed according to the recommendations of the American Thoracic Society/European Respiratory Society using Vmax 22 (Sensor-Medics, Yorba Linda, CA, USA) and PFDX equipment (MedGraphics, St Paul, MN, USA) [28]. Absolute FVC and FEV_1_ values were acquired, and the percentage of their predicted values was calculated from equations obtained using a representative Korean sample [29]. Reversibility was defined as a post-bronchodilator increase in FEV_1_ of at least 12% and 200 mL from the baseline value [16].

St George’s Respiratory Questionnaire (SGRQ) [30] scores were obtained every year during follow-up [26]. AEs were assessed at the clinic every 3 months, where possible. Moderate AE was defined as a clinic visit, while severe AE was defined as hospitalization or an emergency room visit owing to one or more of the following: worsening of dyspnea, increased sputum volume and purulent sputum [31].

### Statistical analyses

Incidence rates for overall mortality were calculated by dividing the number of deaths during the study period by the total observation time. A Poisson regression model was used to estimate the mortality rate ratio (MRR) of the V and PH groups compared to the PL group. Univariable and multivariable analyses were conducted to investigate the association between eosinophil changes and mortality. Multivariable analysis variables included age, smoking status, self-reported history of asthma, two or more moderate exacerbations or one or more severe exacerbations during the previous year, a post-bronchodilator FEV_1_ < 50% predicted at baseline, and use of ICS/long-acting β_2_-agonist (LABA) or ICS for more than two thirds of the study period.

Serial changes in FEV_1_ or the SGRQ score were also compared among the groups using linear mixed models for longitudinal data with random intercepts and random slopes [32]. Models were adjusted to account for potential confounding factors: age, smoking status, self-reported history of asthma, two or more moderate exacerbations or one or more severe exacerbations during the previous year, and use of ICS/LABA or ICS for more than two thirds of the study period. Regarding serial changes in the SGRQ score, a post-bronchodilator FEV_1_ < 50% predicted at baseline was further adjusted.

All tests were two-sided, and *p-*values < 0.05 were considered significant. All analyses were performed using Stata software (ver. 14.0; Stata Corporation, College Station, TX, USA).

## Results

**Fig. 1:**
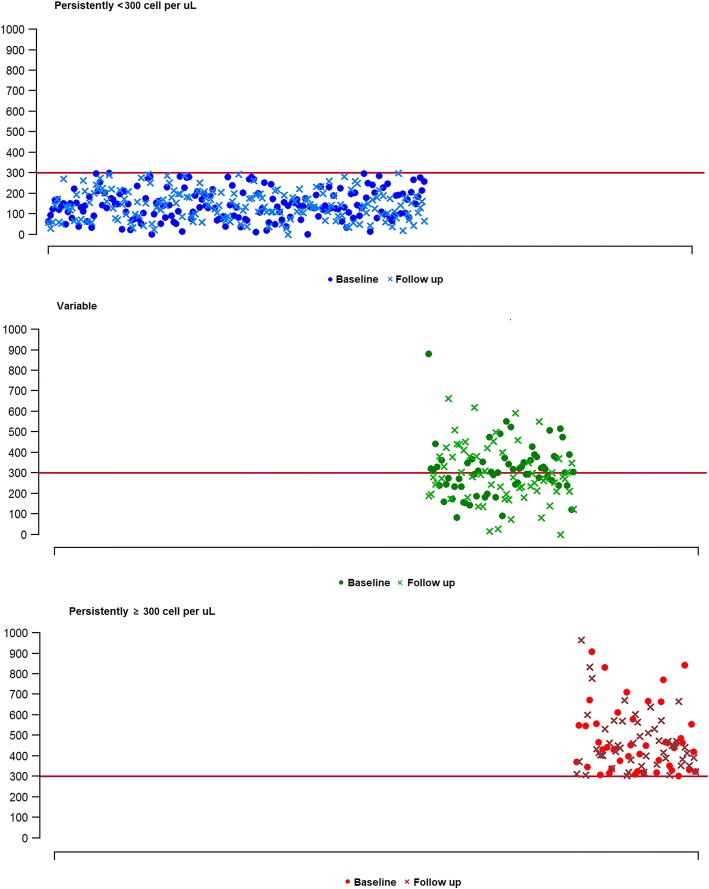
Distribution of blood eosinophils across two serial measurements. Individual patients are shown along the horizontal axis. The top panel shows data for patients with blood eosinophils that were persistently below 300 cells/μL. The middle panel shows data for patients with blood eosinophils that varied around 300 cells/μL. The bottom panel shows patients with blood eosinophils that were persistently equal to or greater than 300 cells/μL. Nine and fourteen participants had counts above 1000 cells/μL in the variable and persistently high groups, respectively (not shown). *The horizontal line at 300 cells/μL is defined as peripheral eosinophilia

The mean (SD) age of the study participants was 66.8 (7.4) years, and the mean duration between eosinophil measurements was 13.3 (4.8) months. Among the 299 COPD subjects, 175 (58.5%), 68 (22.7%), and 56 (18.7%) subjects were stratified into the PL, V, and PH groups, respectively (Fig. 1). The mean (SD) blood eosinophil count at baseline was 143.4 (73.4) cells/μL, 339.0 (191.6) cells/μL, and 681.0 (595.6) cells/μL in the PL, V, and PH groups, respectively. Compared to the PH group, the rate of use of a long-acting muscarinic antagonist was significantly higher in the PL group, but there were no significant differences in terms of age, sex, smoking history, body mass index (BMI), education, symptoms and two or more moderate exacerbations or one or more severe exacerbations during the previous year, self-reported history of asthma, presence of emphysema, or use of ICS/LABA or ICS at baseline (Table 1). The use of ICS or systemic corticosteroids did not differ among groups during the follow-up (Additional file 1: Table S1).

**Table 1:** Baseline characteristics by eosinophil stability

	Persistently < 300	Variable	Persistently ≥300	*P*-value
Number	175	68	56	
Age, years	67.1 (7.6)	66.0 (6.8)	66.6 (7.3)	0.59
Sex				0.18
Female	3 (1.7)	4 (5.9)	1 (1.8)	
Male	172 (98.3)	64 (94.1)	55 (98.2)	
Smoking history				0.28
Current	52 (29.7)	23 (33.8)	23 (41.1)	
Ex-smoker	123 (70.3)	45 (66.2)	33 (58.9)	
BMI, kg/m^2^	22.8 (3.5)	23.5 (2.7)	23.9 (3.0)	0.07
Education				0.63
< high school	86 (49.1)	30 (44.1)	24 (42.9)	
≥ high school	89 (50.9)	38 (55.9)	32 (57.1)	
mMRC ≥2	98 (56.0)	31 (45.6)	28 (50.0)	0.32
SGRQ				
Symptoms	44.1 (19.0)	41.1 (18.8)	46.3 (16.9)	0.39
Activity	48.6 (22.9)	46.0 (24.0)	49.0 (22.3)	0.68
Impacts	22.9 (18.2)	18.2 (15.9)	24.1 (18.8)	0.17
Total	33.7 (17.5)	30.7 (16.6)	35.4 (17.5)	0.29
Acute exacerbation in the previous year^a^				
≥ 2 Moderate AE	15 (8.6)	1 (1.5)	6 (10.7)	0.09
≥ 1 Severe AE	19 (10.9)	6 (8.8)	7 (12.5)	0.80
≥ 2 Moderate or ≥ 1 severe AE	30 (17.1)	7 (10.3)	12 (21.4)	0.23
Self-reported history of asthma	52 (29.7)	17 (25.0)	15 (26.8)	0.74
Spirometry				
FEV_1_ (mL)	1398.7 (510.2)	1538.1 (531.0)	1533.9 (503.1)	0.08
FEV_1_, % predicted	46.5 (15.7)	50.7 (14.8)	49.8 (14.2)	0.10
FVC (mL)	3234.1 (784.1)	3314.3 (800.4)	3240.2 (775.5)	0.77
FVC, % predicted	77.7 (17.6)	79.9 (16.1)	75.8 (15.3)	0.41
FEV_1_/FVC (%)	43.1 (11.2)	45.9 (9.3)	47.3 (10.2)	0.018
Post-bronchodilator FEV_1_ (mL)	1555.6 (531.7)	1690.7 (537.9)	1728.8 (554.1)	0.052
Post-bronchodilator FEV_1_, % predicted	51.6 (16.1)	55.9 (14.9)	56.1 (15.4)	0.06
Post-bronchodilator FEV_1_ < 50%predicted, n(%)	91 (52.0)	42 (61.8)	34 (60.7)	0.28
Positive reversibility, n (%)	54 (30.9)	21 (30.9)	19 (33.9)	0.91
Emphysema > 5%^b^	137 (78.3)	52 (76.5)	43 (76.8)	0.94
Inhaler use				
LAMA	75 (42.9)	22 (32.4)	14 (25.0)	0.036
ICS/LABA or ICS	71 (40.6)	22 (32.4)	28 (50.0)	0.14
Blood Eosinophils	143.4 (73.4)	339.0 (191.6)	681.0 (595.6)	< 0.001
Interval between two Eosinophil count, months	12.2 (4.5)	12.1 (4.0)	12.9 (6.4)	0.56

The data are presented as number (%) or as mean (SD)

Abbreviations: *BMI* body mass index, *mMRC* modified Medical Research Council, *SGRQ* St George’s Respiratory Questionnaire, *AE* acute exacerbation, *FEV*_*1*_ forced expiratory volume in 1 second, *FVC* forced vital capacity, *LAMA* long acting muscarinic antagonist, *ICS* inhaled corticosteroids, *LABA* long acting beta2-agonist, *SD* standard deviation

^a^Moderate AE was defined as a clinic visit and severe AE was defined as a hospitalization or an emergency room visit owing to one or more of the following: worsening of dyspnea, increased sputum volume and purulent sputum

^b^Emphysema was defined as a percentage of lung attenuation less than 950 Hounsfield units. Percent emphysema was determined for total lung

**Fig. 2:**
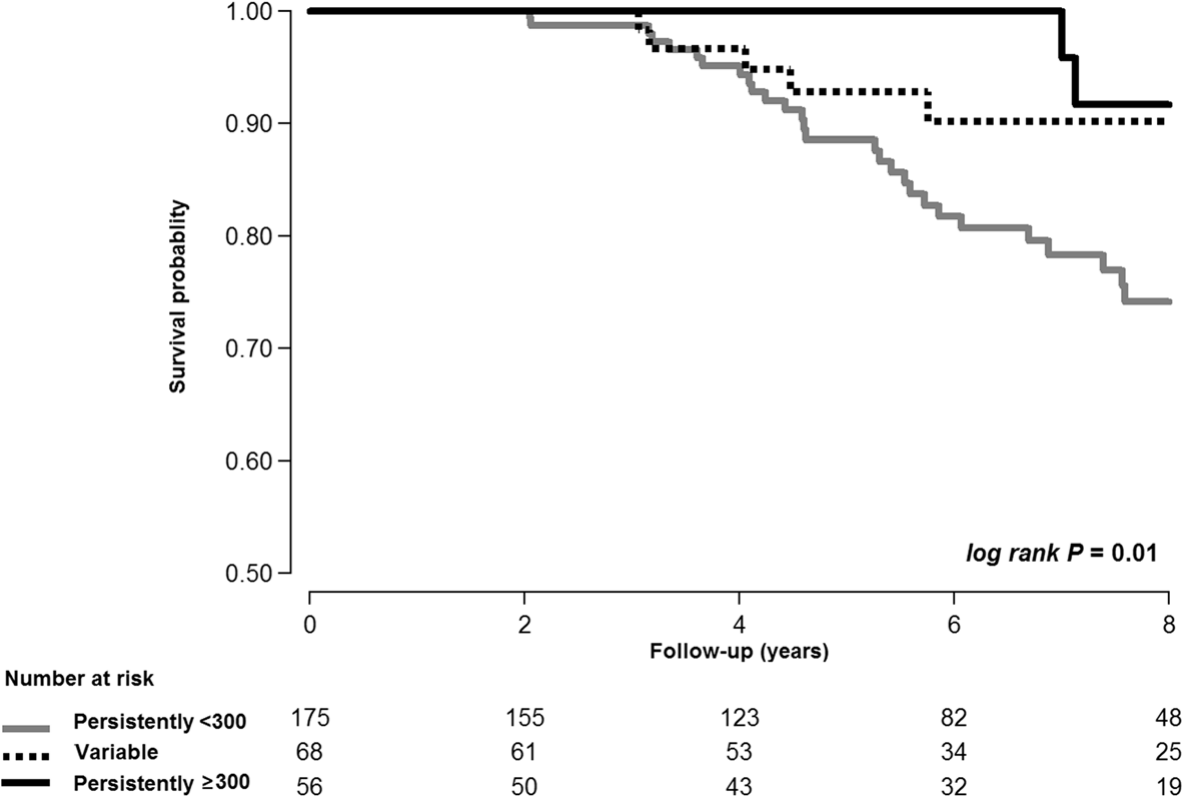
Kaplan-Meier curves for mortality according to blood eosinophil stability

The median follow-up duration was 6.0 years (interquartile range: 3.7–8.0 years), contributing 1649 person-years of follow-up. During follow-up, 28, 5, and 2 subjects in the PL, V, and PH groups died, respectively. The overall mortality rate was significantly lower in the PH group (0.6 [95% confidence interval (CI), 0.2–2.4] per 100 person-years) compared to the PL group (3.0 [95% CI, 2.1–4.3] per 100 person-years) (MRR, 0.27; 95% CI 0.08–0.87; *P* = 0.029; Table 2). The V group had a lower mortality rate than the PL group, but the difference was not significant (MRR, 0.68; 95% CI, 0.32–1.43; *P* = 0.31). After adjusting for age, smoking status, self-reported history of asthma, two or more moderate exacerbations or one or more severe exacerbations during the previous year, post-bronchodilator FEV_1_ < 50% predicted at baseline, and use of ICS/LABA or ICS for more than two thirds of the study period, the PH group still had a significantly lower mortality rate than the PL group (MRR, 0.29; 95% CI, 0.09–0.97, *P* = 0.045) (Fig. 2).

**Table 2:** Association between eosinophil stability and all-cause mortality

	Person-years	No. of cases	Mortality rate (per 100 person-years)	Crude MRR (95% CI)	Adjusted* MRR (95% CI)
Persistently < 300 (n = 175)	935.2	28	3.0	*Reference*	*Reference*
Variable (n = 68)	386.4	5	1.3	0.68 (0.32, 1.43)	0.78 (0.37, 1.66)
Persistently ≥300 (n = 56)	327.6	2	0.6	0.27 (0.08, 0.87)	0.29 (0.09, 0.97)

Abbreviations: *CI* confidence interval, *MRR* mortality rate ratio

*Adjusted for age, smoking status, self-reported history of asthma, two or more moderate exacerbations or one or more severe exacerbation during the previous year, post-bronchodilator FEV_1_ < 50% predicted at baseline, and use of inhaled corticosteroids (ICS)/long-acting β_2_-agonist or ICS for more than two thirds of the study period

Among all participants, the annual change in FEV_1_ was − 29.71 mL in the PL group, − 18.48 mL in the V group, and − 28.15 mL in the PH group. Although the annual change in FEV_1_ was modest in the V and PH groups compared to the PL group, the differences were not significant even after adjusting for age, smoking status, self-reported history of asthma, two or more moderate exacerbations or one or more severe exacerbations during the previous year, and use of ICS/LABA or ICS for more than two thirds of the study period (Table 3). Annually measured SGRQ scores were improved in the PH group compared to the PL group, especially in terms of the symptoms and impact domains (Table 3). However, the presence of two or more moderate exacerbations per year, or of one or more severe exacerbations per year during the study period, did not differ among the groups.

**Table 3:** Change of lung function and SGRQ score over time by eosinophil stability

	Persistently < 300 (*n* = 175)	Variable (*n* = 68)	Persistently ≥300 (*n* = 56)
FEV_1_, mL			
Change of FEV_1,_ mL/year	− 29.71 (− 37.90, − 21.52)	− 18.48 (− 31.03, − 5.92)	− 28.15 (− 41.61, − 14.69)
Difference in change of FEV_1,_ mL/year	*Reference*	11.23 (− 3.76, 26.23)	1.56 (− 14.20, 17.32)
Adjusted^a^ change of FEV_1,_ mL/year	− 29.66 (− 37.92, − 21.41)	−18.42 (− 31.06, − 5.79)	− 28.15 (− 41.70, − 14.60)
Adjusted^a^ difference in change of FEV_1,_ mL/year	*Reference*	11.23 (− 3.86, 26.32)	1.50 (− 14.36, 17.36)
SGRQ score			
Symptoms			
Adjusted^b^ change of score /year	−0.22 (− 1.45, 1.01)	1.45 (− 0.34, 3.24)	−2.75 (− 4.77, − 0.73)
Adjusted^b^ difference in score /year	*Reference*	1.68 (−0.50, 3.85)	−2.53 (− 4.89, − 0.16)
Activity			
Adjusted^b^ change of score /year	0.14 (−1.61, 1.88)	0.23 (− 2.36, 2.81)	−1.07 (− 3.91, 1.77)
Adjusted^b^ difference in score /year	*Reference*	0.09 (−3.03, 3.21)	−1.21 (− 4.54, 2.13)
Impact			
Adjusted^b^ change of score /year	0.73 (−0.62, 2.07)	− 0.24 (− 2.22, 1.75)	−2.44 (− 4.63, − 0.25)
Adjusted^b^ difference in score /year	*Reference*	−0.97(− 3.36, 1.43)	− 3.17 (− 5.74, − 0.60)
Total			
Adjusted^b^ change of score /year	0.26 (−1.15, 1.66)	0.14 (− 1.95, 2.24)	− 2.34 (− 4.63, − 0.04)
Adjusted^b^ difference in score /year	*Reference*	−0.11 (−2.64, 2.41)	− 2.59 (− 5.29, 0.09)

Abbreviations: *SGRQ* St George’s Respiratory Questionnaire, *FEV*_*1*_ forced expiratory volume in 1 second

^a^Adjusted for age, smoking status, self-reported history of asthma, two or more moderate exacerbations or one or more severe exacerbation during the previous year, and use of inhaled corticosteroids(ICS)/long-acting β_2_-agonist or ICS for more than two thirds of the study period

^b^Adjusted for age, smoking status, self-reported history of asthma, two or more moderate exacerbations or one or more severe exacerbation during the previous year, post-bronchodilator FEV_1_ < 50% predicted at baseline, and use of inhaled corticosteroids (ICS)/long-acting β_2_-agonist or ICS for more than two thirds of the study period

## Discussion

In this study, 77% of subjects had stable blood eosinophil count across two serial measurements when a cut-off of 300 cells/μL was used. When patients were stratified into persistently low, variable, and persistently high blood eosinophil groups, no significant differences in baseline characteristics were detected among the groups. During a median follow-up period of 6 years, patients with persistently high blood eosinophils had a better survival rate and improved health-related quality of life measured by SGRQ score than those with persistently low blood eosinophils. However, no significant difference was found in annual lung function decline or exacerbations during the follow-up period among the groups.

The distribution of patients according to blood eosinophil stability in the current study does not agree with the results of the ECLIPSE study, in which only 13.6% of subjects had persistently low blood eosinophils, and 49% and 37.4% had variable and persistently high blood eosinophils, respectively [19]. Given that the stability of eosinophils decreases over time, [25] the use of only two serial measurements (mostly baseline and at 1 year) in the current study would explain the difference between the stability of blood eosinophils reported here and those measured in the ECLIPSE study (where measurements were taken over a 3-year follow-up period). Although the number of subjects significantly decreased as the number of measurements increased in the current study, 47.0% of subject showed persistently low blood eosinophils, and 41.2% and 11.8% had variable and persistently high blood eosinophils among the 170 patients who underwent four measurements of blood eosinophils during follow-up (Additional file 1: Table S2). This proportion was consistent with those from BMI, Obstruction, Dyspnea, Exercise (BODE) index cohort and COPD History Assessment in Spain (CHAIN) cohort, which used a cut-off of 300 cells/μL and three serial measurements [22]. Thus, the difference in blood eosinophils stability most likely arises from the ECLIPSE cut-off value of 2%, which is surpassed in 57–75% of COPD patients [11, 33]. Such a low threshold for eosinophils might have diluted the differences within eosinophilic inflammation, similar to the way in which the benefit of ICS for exacerbation prevention becomes more obvious as the eosinophil threshold rises [8–10, 34]. In addition, the relative percentage using the total leukocyte count is thought to be less reliable than the absolute blood eosinophil count [35]. Thus, a higher threshold of 300 cells/μL was used in this study for the absolute eosinophil count based on previous studies [10, 20, 27] and expert consensus on the asthma-COPD overlap [36].

Using an absolute eosinophil count of 300 cells/μL from two serial measurements as the cut-off, the current study found that patients with persistently high blood eosinophils had a better survival rate than those with persistently low blood eosinophils. These results are consistent with those of recent studies that used at least three measurements of blood eosinophils [22, 23]. In a study using data from the BODE cohort, patients with blood eosinophils persistently lower than 300 cells/μL over 2 years (three measurements) had an increased risk of all-cause mortality [adjusted hazard ratio, 7.287 (95% CI, 1.004–52.891); *p* = 0.050] [22]. Another study revealed that patients with blood eosinophils persistently ≥150 cell/μL over 5 years had a better survival rate than other patients (*p* < 0.01) [23]. A study using only one eosinophil measurement in patients with severe exacerbation found that eosinopenia (< 50 cell/μL) was associated with increased in-hospital mortality [odds ratio, 2.76 (95% CI, 1.58–4.83), *p* = 0.001] [37]. Data from the Hokkaido COPD cohort showed that COPD patients with two or more asthma-like features (including a baseline blood eosinophil count ≥300 cells/μL) had significantly lower 10-year all-cause mortality (*p* = 0.020) than other patients, but baseline eosinophilia alone did not reach statistical significance for all-cause mortality [20]. Given that the V group in the current study did not differ significantly from the PL group in terms of mortality, but the PH group had significantly lower mortality, at least two eosinophil counts appear to be necessary to identify patients with different clinical courses.

This study also found that patients with persistently high blood eosinophils had an improved SGRQ score, especially in terms of the symptoms and impact domains of the SGRQ, than those with persistently low blood eosinophils. However, although there was a trend toward a reduced rate of FEV_1_ decline in the V group, those groups with persistently low, variable, and persistently high blood eosinophils showed no difference in annual lung function decline. This finding is consistent with that of the ECLIPSE investigators, who found no significant differences in the rate of decline in FEV_1_ according to blood eosinophil pattern using a cut-off value of 2%. In a re-analysis of the Inhaled Steroids in Obstructive Lung Disease (ISOLDE) trial, ICS treatment led to a reduced rate of FEV_1_ decline in COPD patients with a baseline blood eosinophil count of ≥2% compared to those with a baseline blood eosinophil count of < 2% [7]. In the current study, the proportion of users of ICS treatment was similar between the groups (Additional file 1: Table S1), which might contribute to a lack of signals for annual lung function decline.

The present study had several limitations. First, this study defined COPD using a fixed airflow limitation with a smoking history of more than 10 pack-years and 97% of the subjects were male, which could limit the generalisability to non-smoking and/or female patients. In Korea, the prevalence of COPD is four times lower in females compared to males and this gender discrepancy is largely due to the very low smoking rate among females [38, 39]. Second, the results of this study should be interpreted in light of its retrospective nature and prospectively collected data, as well as the relatively small sample size, despite the long median follow-up duration. Third, an optimal interval between blood eosinophil measurements necessary to establish a patient’s blood eosinophil count as persistently high was unable to be determined. Further studies are necessary to determine whether those with persistently high eosinophils during short-term follow-up (i.e., 6 months) have consistent results. Finally, since blood eosinophils were unable to be compared to those of sputum eosinophils or other inflammatory markers (e.g., IL-5, IL-13) in the study subjects, there was limited evidence to support the biologic plausibility of these findings.

In conclusion, COPD patients with persistently high blood eosinophils had a better survival rate and improved symptoms and impact domains of SGRQ score than those with persistently low blood eosinophils, while those with variable blood eosinophils had survival rates similar to those with persistently low blood eosinophils. Two serial measurements of blood eosinophils could therefore help predict the outcomes of COPD patients, but this finding should be validated in a future study with a larger number of subjects.

## Additional file

Additional file 1: **Table S1.** Use of ICS containing inhalers or systemic corticosteroids during the follow-up period. **Table S2.** Stability of blood eosinophils in patients with blood eosinophils measured more than two times. (DOCX 21 kb)

## Abbreviation

COPD: chronic obstructive lung disease
FEV_1_: forced expiratory volume in 1 second
FVC: forced vital capacity
ICS: inhaled corticosteroids
KOLD: Korean Obstructive Lung Disease
LABA: long-acting β_2_-agonist
MRR: mortality rate ratio
PH: persistently high
PL: persistently low
V: variable
