# Supplementary material for: Serial blood eosinophils and clinical outcome in patients with chronic obstructive pulmonary disease
Source: Respir Res. 2018 Jul 13;19:134. doi: 10.1186/s12931-018-0840-x (PMC6044087; doi:10.1186/s12931-018-0840-x)
Supplement: Supplementary file 1 — Table S1. Use of ICS containing inhalers or systemic corticosteroids during the follow-up period. Table S2. Stability of blood eosinophils in patients with blood eosinophils measured more than two times. (DOCX 21 kb) [file 12931_2018_840_MOESM1_ESM.docx]

**Table S1** Use of ICS containing inhalers or systemic corticosteroids during the follow-up period

|  | **Persistently < 300**  **(n= 175)** | **Variable**  **(n= 68)** | **Persistently ≥ 300**  **(n= 56)** | ***P*-value** |
| --- | --- | --- | --- | --- |
| Use of ICS/LABA or ICS^*^ | 106 (60.6) | 46 (67.7) | 39 (69.6) | 0.36 |
| Use of systemic corticosteroids^†^ | 59 (33.7) | 24 (35.3) | 13 (23.2) | 0.28 |

Data are presented as number (%)

Abbreviations: ICS, inhaled corticosteroids; LABA, long-acting β2-agonist

^*^ Defined as when the ICS/LABA or ICS was prescribed for more than two thirds of the study period.

^†^ Defined as when the systemic steroid was prescribed at least once during the study period.

**Table S2** Stability of blood eosinophils in patients with blood eosinophils measured more than two times

|  | **Persistently < 300** | **Variable** | **Persistently ≥ 300** |
| --- | --- | --- | --- |
| **Two measurements (n=299)** | 175 (58.5) | 68 (22.7) | 56 (18.7) |
| **Three measurements (n=231)** | 116 (50.2) | 87 (37.7) | 28 (12.1) |
| **Four measurements (n=170)** | 80 (47.0) | 70 (41.2) | 20 (11.8) |

Data are presented as number (%)
